# *Abhraka Bhasma* (mica based nanomedicine): an ayurvedic herbomineral perspective in breast cancer management

**DOI:** 10.3389/fphar.2025.1656846

**Published:** 2025-12-19

**Authors:** Dhanya Soman Pillai, Amarnath Karavettekudy Ranjit

**Affiliations:** 1 Department of Rasashastra and Bhaishajya Kalpana (Pharmaceuticals), Amrita School of Ayurveda, Amritapuri, Amrita Vishwa Vidyapeetham, Kollam, Kerala, India; 2 Department of Computer Science and Engineering, Amrita school of Computing, Amritapuri, Amrita Vishwa Vidyapeetham, Kollam, Kerala, India

**Keywords:** Abhraka bhasma, breast cancer, alternative medicine, immunomodulation, genotoxicity

## Abstract

**Background:**

Abhraka Bhasma (Mica nanoparticles) is an Ayurvedic herbomineral medicine traditionally used in the management of conditions similar to breast cancer. Its rationale is based on its *Dhatu-Pushtikara* (tissue-nourishing), *Rasayana* (rejuvenating) and *Tridosha*-balancing properties, suggesting its potential for evaluation in integrative oncology.

**Objective:**

This work aims to examine the therapeutic potential of Mica nanoparticles (*Abhraka Bhasma*) as an alternative medicine in the management of breast cancer.

**Methods (type of evidence):**

This mini-review analyses the preclinical and limited clinical evidence supporting *Abhraka Bhasma* (mica nanoparticles) as a potential adjunct in breast cancer management. The mechanistic basis was evaluated from *in vitro* and *in vivo* models.

**Key findings:**

*In vitro*: *Abhraka Bhasma* exhibits dose-dependent cytotoxicity, apoptosis, immunomodulatory activity and inhibition of teratoma-formation in different cell lines. *In vivo*: studies support these findings, indicating enhanced DNA repair capacity, reduced genotoxicity, chemopreventive responses, immunostimulatory effects and modulation of oxidative stress.

**Conclusion:**

The traditional Ayurvedic rationale for *Abhraka Bhasma* correlates with reported preclinical mechanisms. Thus, the ancient wisdom and modern evidence make *Abhraka Bhasma* an important part of integrative oncology, which offers a complementary strategy to improve patient outcomes. Available evidence on *Abhraka Bhasma* in cancer treatment is currently preclinical data (Level 5) and hypothesis generating only. To date, no RCTs or cohort studies (Levels 1–3) on the safety and efficacy of *Abhraka Bhasma* as an adjunct in breast cancer treatment have been published. To bridge the gap between traditional use and evidence based clinical application, a structured and systematic research pathway is essential.

## 1 Introduction

According to the GLOBOCAN 2020 estimates, breast cancer has become the most frequently diagnosed cancer worldwide, with over 2.26 million new cases and nearly 685,000 deaths reported in 2020 (Sung et al., 2021). This makes it a leading global health challenge. In earlier days mastectomy followed by chemotherapy was the standard treatment for breast cancer. At the same time, they have limitations such as negative impact on quality of life, drug resistance and toxicity (Zafar et al., 2025). These challenges shifted researchers to integrative approaches and novel therapies like biocompatible nanomedicines. The traditional Ayurvedic medicine *Abhraka bhasma* which consists of mica nanoparticles has been identified as an alternative medicine and potential adjunct for cancer therapy.

*Bhasma*s are Ayurvedic nano medicines prepared by processing metals and minerals with herbal products to eliminate their toxicity and confer therapeutic properties (Kantak et al., 2020; Sharma, 2004; Acharya, 2015; Government of India, 2003; Wele et al., 2021). As the particles in the final product are reportedly within the nanometer range, they are considered nanomedicines (Kantak et al., 2020). *Abhraka Bhasma* an incinerated herbo-metallic preparation of mica, is widely used by traditional medicine practitioners (Chatterjee et al., 2024) for managing *Arbuda* (tumors). This is based on its traditionally attributed properties, such as *Rasayana* (rejuvenating), detoxifying nature, and immune-supportive properties.

Breast cancer, the most frequently diagnosed cancer worldwide, is a leading global health challenge. In Ayurveda, Breast cancer (*Stanarbuda*) is attributed to a *tridosha* (vital forces) imbalance causing abnormal *Mamsa Dhatu* (muscle tissue proliferation). The rationale for using Abhraka Bhasma is that its properties counter this pathology. Its Madhura rasa (Sweet taste) and Sheeta virya (cold nature) make it a Rasayana (rejuvenator) to restore Ojas (immunity). It is Tridoshahara (balancing doshas), and its Lekhana (scraping) Karma is traditionally thought to clear the abnormal Mamsa Dhatu proliferation.

## 2 Abhraka Bhasma

### 2.1 Mechanism of preparation and composition of *Abhraka Bhasma*

**FIGURE 1:**
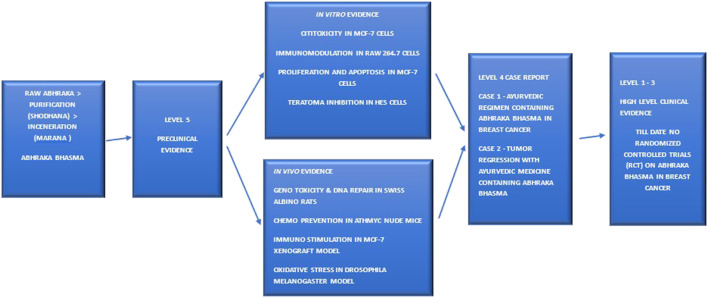
A Schematic framework illustrating the preparation, characterization, and evidence levels for *Abhraka Bhasma* in cancer.

*Abhraka Bhasma* is a nanoparticle-based medicine derived from raw mica (Kantak et al., 2020). Its preparation involves traditional pharmaceutical processes, primarily *shodhana* (purification) followed by numerous cycles of *puta* (incineration) (4, p. 474–476, 5, p. 67–69, 5, p. 65–72, 7). The *puta* (calcination) is the key nano-transformation step (4, p. 476–495, 5, p. 68–72, 7, 8, 9), designed to convert the raw material into a non-toxic, bioavailable, and therapeutically potent medicine (Figure 1). Analytical techniques confirm the final composition consists of oxides of iron, silica, alumina, magnesium, and potassium, with a reduced nanoparticle size of 20–100 nm (Kantak et al., 2020; Wele et al., 2021) (Table 1).

**TABLE 1:** Comparitive Analysis of *Abhraka Bhasma* and Biomedical Mica (STB- HO), Quantitative Toxicology of *Abhraka Bhasma* and Safety profile.

Parameters	*Abhraka Bhasma* (mica nanoparticles)	STB- HO (biomedical Mica)	References
Preparation	A series of pharmaceutical processes involving purification (shodhana), and incineration (marana)	Mica is purified and physically processed by dry cutting or milling techniques to get small particle size	Kantak et al. (2020), Cho et al. (2013)
Composition	KMg_3_(Si_3_Al)O_10_(OH)_2_	Alumino silicate	Kantak et al. (2020), Wele et al. (2021)
Elemental composition	Si(14.18%), Al (7.58%), Mg (5.03%), Fe (12.64%), K (8.36%), Na (1.39%), Rb, Ca (3.64%), O (35.69%), C (9.57%), Ti (1.16%), Cs, P (0.76%), Mn, Cr, Li, Ba		Kantak et al. (2020), Wele et al. (2021)
Particle size distribution	SEM - 92.3 nm TEM – 50 nm to 1 µm XRD – 62 nm	Nanometer range	Kantak et al. (2020), Wele et al. (2021)
Dosing	120 mg–360 mg/kg body weight	100 mg/kg body weight (animal models)	Kulala et al. (2023), Cho et al. (2013)
Toxicity outcomes with methods and acute/sub- acute metrics	Low acute toxicity but at higher doses reported to have reversible, dose-dependent hepatotoxicity, No genotoxicity	No adverse effects like loss of body weight or abnormal behaviour, reported in animal models suggesting low toxicity profile *in vivo*	Cho et al. (2013), Gopinath and Shivashankar (2021), Pandit et al. (2024)
Toxicity data of abhraka bhasma	*Acute toxicity - LD50 as per OECD guidelines in rat model shows no toxicity upto 2000 mg/kg AB orally for 2 weeks*Sub-acute toxicity of different AB doses showed no significant variation when compared with the control		Gopinath and Shivashankar (2021)
	Low acute toxicity but at higher doses reported to have reversible, dose-dependent hepatotoxicity		Pandit et al. (2024)
	*Sub-acute toxicity – NOAEL, as per OECD 407, for 28-days, in Sprague-Dawley rats upto 1280 mg/kg b.w.a formulation containing AB, shows no changes in the body weight, organ weight & haematologicaical parameters		Balakrishna et al. (2025)
	* Genotoxicity, in swiss albino rats at a dose of 120 mg/kg or 360 mg/kg body weight - nongenotoxic, enhance DNA repair capacity		Kulala et al. (2023)
Safety data (International Council for Harmonisation ICH, 2022)			
Metal regulatory limit Mercury (Hg) - 30 μg/day Arsenic (as) - 15 μg/day Lead (Pb) - 5 μg/day			

### 2.2 Mechanism of action of *Abhraka Bhasma*

Mechanism of Action of *Abhraka Bhasma* against breast cancer is twofold. One demonstrates direct, dose-dependent cytotoxicity, inhibiting the proliferation of MCF-7, human breast cancer cells. Simultaneously, it exhibits significant immunomodulatory and anti-inflammatory effects by reducing nitric oxide production in the tumor microenvironment. *Abhraka Bhasma* is believed to trigger apoptosis, effectively targeting cancer cells while also regulating the surrounding immune response.

### 2.3 Quantitative toxicology data from standardized *Abhraka Bhasma*

For the clinical translation of a Bhasma, the primary concern is safety (Kulkarni et al., 2024), which is directly connected to proper pharmaceutical preparation, heavy-metal content, and batch-to-batch consistency.

#### 2.3.1 LD50 and NOAEL

Preclinical toxicity studies indicate *Abhraka Bhasma* has low acute toxicity, with an LD50 > 2000 mg/kg in rats, classifying it as non-toxic per OECD guidelines (Gopinath and Shivashankar, 2021). However, a 28-day sub-acute study suggested reversible, dose-dependent hepatotoxicity at higher doses, requiring careful dosage (Pandit et al., 2024). In contrast, another subacute study on a formulation containing it established a NOAEL >1000 mg/kg/day with no toxicologically relevant changes (Balkrishna et al., 2025). Furthermore, it was found to be non-genotoxic and to enhance DNA base excision repair capacity in mice (Kulala et al., 2023) (Table 1).

#### 2.3.2 Elemental profile and heavy-metal risk

Quantitative analysis of standardized *Abhraka Bhasma* indicates a typical elemental profile. A study by Wele et al., reported the elemental constitution of *Abhraka Bhasma* as Si(14.18%),Al (7.58%),Mg (5.03%),Fe (12.64%),K (8.36%),Na (1.39%),Ca (3.64%),O (35.69%), C (9.57%),Ti (1.16%), P (0.76%),Mn, Cr, Li, Ba, Rb, Cs (Kantak et al., 2020; Wele et al., 2021). A study by Bhatia et al., (Bhatia and Kale, 2013), reported the absence of toxic heavy metals like Mercury and organic compounds in *Abhraka Bhasma*. But the high percentage of aluminum (Al) in the formulation is a notable finding that warrants further toxicological assessment (Table 1).

#### 2.3.3 Bioaccessibility

Bioaccessibility is the amount of a nutrient or compound that is released during digestion and made available for absorption in the small intestine. It is a crucial step in the overall process of bioavailability. A study conducted by Kantak et al., revealed that the bio-accessibility of elements like K, Ca, Al, Fe, Na and Si in *Abhraka Bhasma* is greater in gastric digestion than in gastro-intestinal digestion (Kantak and Rajurkar, 2023).

#### 2.3.4 Batch variability

Batch-to-batch variability, stemming from different source materials or processing, is a key challenge for standardizing *Abhraka Bhasma*. Kantak et al. (2020), compared two traditional preparation methods and two commercial samples, finding that different methods yield physically different end-products (e.g., varying nanoparticle proportions), which they hypothesized could correlate with efficacy. This demonstrates that manufacturing differences make it difficult to generalize safety, toxicity, or efficacy data from a single preparation.

#### 2.3.5 Pharmacovigilance considerations

Any future clinical application would necessitate not only stringent ethical approval but also robust pharmacovigilance systems to proactively monitor for potential adverse effects, particularly those related to its elemental composition. This post-marketing surveillance is an essential regulatory requirement for ensuring patient safety during any potential clinical integration.

### 2.4 Distinguishing traditional *Abhraka Bhasma* from STB HO-modern biomedical mica

Abhraka Bhasma prepared by traditional Ayurvedic methods and STB HO, Biomedical Mica are both described as Mica nanoparticles, but they are prepared by distinctly different methods. The preparation of *Abhraka Bhasma* involves a series of pharmaceutical processes including purification (*Shodhana*), and incineration (*Marana*) which are intended to transform raw mica into a non-toxic, nanoparticle-based medicine (Kantak et al., 2020). *Shodhana* involves repeated heating and quenching of *Abhraka* in liquid media such as milk or herbal decotions. This is followed by *marana* which involves repeated incinerations after trituration with herbal juices. Studies on *Abhraka Bhasma* have reported its anti-tumor, immunomodulatory, anti-inflammatory, antioxidant, cell regeneration, DNA repair mechanisms and immunostimulatory actions (Kantak et al., 2020; Sharma, 2004; Acharya, 2015; Wele et al., 2021; Pandey et al., 2022; Jani et al., 2021) (Table 1).

In contrast, formulations like STB-HO are purified mineral preparations. They are typically manufactured using modern mechanical or physical methods (dry cutting techniques or milling) to achieve a fine particle or nanoparticle size. This process results in nanoparticles, but lacks the repeated incineration and incorporation of herbal-derived organic components seen in the *bhasma* preparation. STB-HO is a medical-grade aluminosilicate mineral derivative in biomedical research. It is a processed form of the natural mineral mica (an aluminosilicate mineral), converted into fine or nanoparticles. Studies on STB-HO, have demonstrated Immunostimulatory, chemopreventive and teratoma prevention action (Kang et al., 2015; Cho et al., 2013; Choi et al., 2016). A primary limitation is the scarcity of detailed, publicly available information regarding STB-HO’s precise manufacturing, standardization, and physicochemical characterization, which hinders direct, rigorous comparisons with traditional *Abhraka Bhasma* and limits a full understanding of its distinct pharmacological profile.

## 3 Anticancer potential: preclinical *In vitro* evidence (level 5)

**FIGURE 2:**
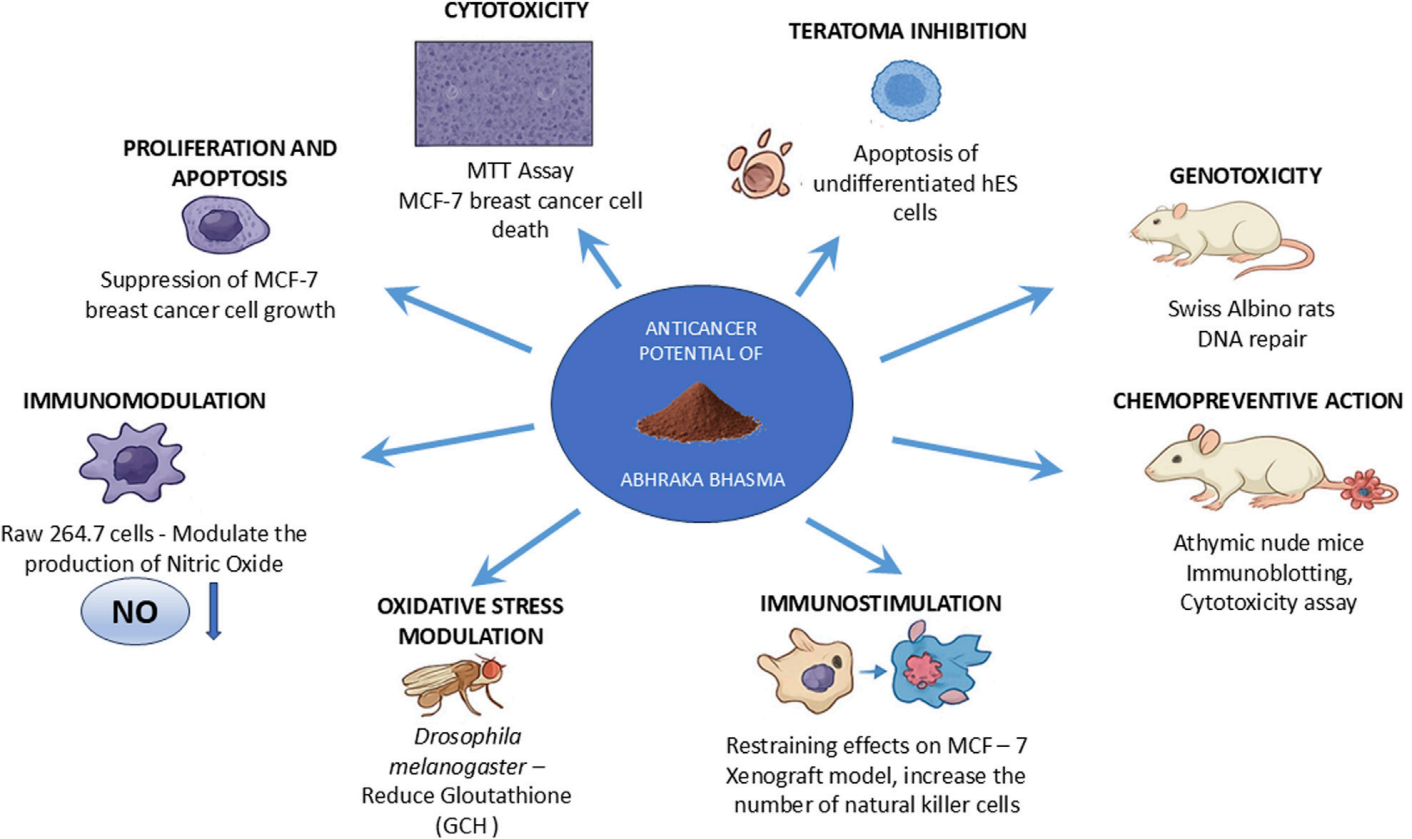
Anticancer potential of *Abhraka Bhasma* (Mica nanoparticles).

*In vitro* evidence for the anticancer potential of *Abhraka Bhasma* and STB HO (Biomedical Mica nanoparticles) is shown in (Figure 2) (Table 2).

**TABLE 2:** Per-study extraction.

Material	Model	Dose	Exposure	Controls	Statistics	Main outcomes	Limitations
Abhraka bhasma (Sreelakshmi and Nandagopalan, 2024)	MCF-7 breast cancer cells	10, 50, 100, 200 μg/mL	48 h	0.1% DMSO		Dose-dependent cytotoxicity (49.22% cell death at 200 μg/mL) and apoptotic morphology	Lack of selective toxicity
Abhraka bhasma (Sreelakshmi and Nandagopalan, 2024)	RAW 264.7 cells (LPS-stimulated)	50, 100, 200 μg/mL	A defined incubation period	LPS (1 μg/mL) treated cells	Significant reduction	Dose-dependent decrease in nitrite levels, suggesting immunomodulatory effects	True effect unclear-whether effect or toxicity?
Shataputi abhraka bhasma (Tamhankar and Gharote, 2020)	Lung (HOP-62), leukemia (U-937), prostate (DU-145) cells	10, 20, 40, 80 μg/mL		Adriamycin (positive control)	Statistically highly significant	Anticancer activity on all three cell lines, especially prostate (DU-145)	
Mica nanoparticle STB-HO (Kang et al., 2015)	MCF-7 cells; co-culture with macrophages, DCs, and NK cells	10 μg/mL and 50 μg/mL	72 h (proliferation); 48 h (macrophages); 4 h (NK co-culture)	Untreated cells; Unstimulated controls	p < 0.001 (macrophages); p < 0.01 (NK cells)	No direct cytotoxicity, but significant immunomodulation (M1 macrophage skewing, NK cell activation)	No statistically significant direct effect
STB-HO mica fine particle (Choi SW et al., 2016)	hES cells; *in vivo* teratoma xenotransplantation	*In vitro*: 1, 10, 100 μg/mL; in vivo: 10 μg/mL pre-treatment	*In vitro*: 24 h (pre-treatment)	Untreated cultures; vehicle treated group	p < 0.05 (*in vitro*); “statistically significant” (*in vivo*)	Selectively eliminated undifferentiated hES cells; pre-treatment prevented teratoma formation (0/15 vs. 10/10)	Specific to undifferentiated hES cells
Abhraka bhasma (Kulala et al., 2023)	Swiss albino mice	120 mg/kg or 360 mg/kg	Orally, daily for 7 days		p > 0.05 (no toxicity); p < 0.05 (protection)	Not genotoxic; protected against EMS-induced chromosomal damage	Dose-dependent toxicity at high concentrations
Particled mica (STB-HO) (Cho et al., 2013)	BALB/c athymic nude mice (HCT116 xenograft)	50 mg/kg or 100 mg/kg	Orally for 41 days	Untreated control	p < 0.05	Significantly suppressed final tumor weight at 100 mg/kg	No direct cytotoxicity
Mica nanoparticle (STB-HO) (Kang et al., 2015)	MCF-7 xenograft model (athymic mice)	35 mg/kg or 70 mg/kg	Orally, daily for 12 weeks	Saline-treated control	p < 0.05; p < 0.01	Significantly reduced final tumor volume and mass; increased splenic NK cells	No direct cytotoxicity
Abhraka bhasma (Subedi et al., 2018a)	*Drosophila melanogaster*	Diet supplemented	Larval and adult stages		Significantly lowered	Modulated SOD/catalase; decreased GSH (∼40–70% in larvae, 31%–36% in adults); increased CncC and Hsp70 gene expression	Translational gap between the *Drosophila* model and human physiology

### 3.1 *Abhraka Bhasma* (mica nanoparticles)

#### 3.1.1 Cytotoxicity on MCF-7 breast cancer cells

Sreelakshmi and Nandagopalan (2024) reported a dose-dependent cytotoxicity (Van Meerloo et al., 2011) of *Abhraka Bhasma* in MCF-7 cells (Lee et al., 2015). It has a high IC50 value of 193.2 μg/mL indicating weak potency (Sreelakshmi and Nandagopalan, 2024). The authors cited morphological observations such as cell shrinkage as evidence of an apoptotic anticancer effect. But these findings are not definitive. Such morphological changes are only suggestive and lack the necessary molecular validation to confirm the pathway. The most significant drawback of this study is the lack of selective toxicity. The study also assessed toxicity against a healthy macrophage cell line (RAW 264.7) and found that the 200 μg/mL dose killed 46.15% of these healthy cells (Sreelakshmi and Nandagopalan, 2024). This demonstrates a complete absence of a therapeutic window, as the cytotoxicity against the healthy cell line (46.15%) is nearly identical to that against the cancer cell line (49.22%) at the same concentration.

#### 3.1.2 Immunomodulatory activity on RAW 264.7 cells

Sreelakshmi and Nandagopalan (2024) evaluated *Abhraka Bhasma* in different concentrations (50, 100, 200 μg/mL) on LPS-stimulated RAW 264.7 cells (Taciak et al., 2018), measuring nitrite, a key inflammatory molecule (Sreelakshmi and Nandagopalan, 2024). The study reported a dose-dependent decrease in nitrite levels (Sreelakshmi and Nandagopalan, 2024; Lepoivre et al., 1990; Gavrilas et al., 2019), which suggest a specific anti-inflammatory effect of the drug, possibly by inhibiting iNOS (Coleman, 2001). The same study demonstrated 46.15% cell death in this cell line at the 200 μg/mL concentration (Sreelakshmi and Nandagopalan, 2024). This high toxicity makes it impossible to analyse specific pathway inhibition from a simple reduction in viable, nitrite-producing cells. The observed anti-inflammatory effect is the expected outcome when nearly half the cell population is non-viable. Though these findings suggests that *Abhraka Bhasma* has an immunomodulatory effect on RAW 264.7 cells, it is unclear if this finding represents a true immunomodulatory effect or is simply a consequence of *Abhraka Bhasma* being toxic to the macrophage cells at the tested concentrations (Sreelakshmi and Nandagopalan, 2024).

#### 3.1.3 Anticancer activity of *Shataputi Abhraka Bhasma* (100 times incinerated mica)

In a study conducted by Tamhankar YL et al. on *Shataputi Abhraka Bhasma* containing mica nanoparticles, incinerated 100 times, various concentrations (10 μg/mL, 20 μg/mL, 40 μg/mL, 80 μg/ML) of *Shataputi Abhraka Bhasma* were introduced into three cancer cell lines and compared against positive control drug Adriamycin. (Tamhankar and Gharote, 2020; Sharma, 2004). This *in vitro* study demonstrated dose-dependent cytotoxicity, with the highest potency observed against the prostate (DU-145) cell line (GI50 = 31.2 μg/mL) compared to lung (HOP-62) and leukemia (U-937) cells (Tamhankar and Gharote, 2020). (Tamhankar and Gharote, 2020). While this paper was limited to a cytotoxicity screen and did not investigate the causal mechanism, it is hypothesized that the anticancer activity is likely attributable to the mica nanoparticles penetrating the cancer cells, where they induce high levels of Reactive Oxygen Species (ROS), leading to overwhelming oxidative stress and the activation of the apoptotic cell death pathway.

### 3.2 STB -HO (Biomedical Mica)

#### 3.2.1 Proliferation and apoptosis in MCF-7 breast cancer cells

An *in vitro* study tested Mica Nanoparticle STB-HO (10 and 50 μg/mL) on MCF-7 cells for 72 h, using untreated cells as a control (Kang et al., 2015). While STB-HO had no statistically significant direct effect on MCF-7 proliferation or apoptosis. (Ebihara et al., 2023). However, STB-HO demonstrated potent indirect anti-tumor activity by activating immune cells. It significantly polarized macrophages toward an immunostimulatory M1 phenotype (P < 0.001) after 48 h (Kang et al., 2015). Furthermore, in a 4-h co-culture with NK cells, STB-HO at (10 μg/mL and 50 μg/mL) generated IFN-γ–secreting effector cells (Tau and Rothman, 2001), causing a statistically significant (P < 0.01) increase in immune cells that can kill MCF-7 cells (Tau and Rothman, 2001). STB-HO functions as an immunomodulator rather than a direct cytotoxic agent, suppressing MCF-7 cells indirectly by polarizing macrophages toward an anti-tumor M1 phenotype and stimulating IFN-γ–secreting NK cells.

#### 3.2.2 Inhibition of teratoma-forming ability in hES cells

In a study conducted by Choi SW et al., the anti-tumorigenic activity of STB-HO Mica fine particle in hES cells (Human Embryonic Stem Cell) (Park et al., 2024) was investigated by administering different concentrations (1, 10, and 100 μg/mL) of Mica fine particles into hES cell (Human Embryonic Stem Cell) (Park et al., 2024) and compared against untreated control cultures (Choi et al., 2016). The study found that STB-HO treatment in differentiating cultures selectively induced apoptosis only in the remaining undifferentiated hESCs, without harming the differentiated cells. This mechanism was p53-dependent. At 10 μg/mL, it activated p53, p21, and pro-apoptotic proteins (Bim, Puma, p-Bad) (Jourdan et al., 2009) while suppressing the anti-apoptotic gene BIRC5 (Gavrilas et al., 2019). This selective elimination of pluripotent cells was validated *in vivo.* hES cells pre-treated with 10 μg/mL STB-HO were incapable of forming teratomas upon xenotransplantation (0/15 animals), a statistically significant contrast to the 100% teratoma formation (10/10 animals) in controls. These findings suggest that pre-treatment of differentiating hES cell cultures with STB-HO can prevent teratoma formation after stem cell transplantation (Choi et al., 2016). However, the mechanism of p53-dependent apoptosis was highly specific to undifferentiated hES cells during the differentiation process.

## 4 Anticancer potential: preclinical *In vivo* evidence (level 5)

*In vivo* evidence for the anticancer potential of *Abhraka Bhasma* and STB HO (Biomedical Mica nanoparticles) is shown in (Figure 2; Table 2).

### 4.1 Genotoxicity and DNA repair potential in swiss albino rats

Following OECD guidelines, an acute oral toxicity study was conducted by Kulala et al., in a Swiss albino mouse model (n = 3) (Kulala et al., 2023). The dosing regimen consisted of 120 mg/kg or 360 mg/kg body weight of *Abhraka Bhasma* administered orally daily for 7 days. The study (n = 6) confirmed *Abhraka Bhasma* was neither genotoxic (micronucleus assay) nor reproductively toxic (sperm abnormality assay) (Kulala et al., 2023). Significantly, it demonstrated a potent chemoprotective role, reducing (p < 0.05) chromosomal damage induced by the mutagen ethyl methanesulfonate. This protection was mechanistically linked to an enhanced constitutive DNA base excision repair (BER) capacity, as validated by an *in vitro* comet-based repair assay (Kulala et al., 2023). This suggests the formulation bolsters intrinsic DNA repair pathways. Even though the study results suggested it to be nongenotoxic, it also provides evidence of dose-dependent toxicity at high concentrations, which was reflected as mild histopathological (liver and kidney) changes observed at the 360 mg/kg dose.

### 4.2 Chemopreventive action on athymic nude mice

Rajput et al. (2008), Cho et al. (2013) examined Mica (STB-HO) in a colorectal cancer Xenograft model (n = 6) (HCT116 cells). Oral administration of 100 mg/kg significantly suppressed final tumor weight (p < 0.05). Subsequent *in vitro* mechanistic studies elucidated the pathway. Mechanistic studies like immunoblotting, cytotoxicity assay, FACs analysis and measurement of matrix metalloproteinase 9 (MMP-9) secretion were also performed on HCT116 and human umbilical vein endothelial cells (HUVECs) for 24 h (Duranova et al., 2024). However, a critical finding from the study is that this anti-tumor effect was not due to direct cytotoxicity of the drug, but was attributed to Cytostatic Effects: Inducing G1 arrest and Anti-angiogenic Effects: inhibiting VEGFR2. The G1 arrest was mediated by the upregulation of p21 and p27, while the anti-angiogenic effect was linked to the suppression of VEGF-induced VEGFR2 phosphorylation (Prasetiyo and Wahjoepramono, 2024), effectively halting both proliferation and the tumor’s blood supply.

### 4.3 Immunostimulatory effects on MCF-7 xenograft model

A study by Kang et al. (2015) in an MCF-7 xenograft model (n = 5, NOG mice co-injected with human PBMCs) found oral STB-HO (35 and 70 mg/kg) significantly reduced final tumor volume and mass (p < 0.01) over 12 weeks (Kang et al., 2015). A critical clarification from the study is that this anti-tumor effect was not due to direct cytotoxicity. This *in vivo* outcome was validated by *in vitro* co-culture assays, where STB-HO drove the activation and expansion of IFN-γ–secreting effector cells. Therefore, the anti-tumor effect of STB-HO is attributed to an indirect immunostimulatory mechanism, which suppresses tumor growth by enhancing the host’s innate immune response, specifically via a significant expansion of the human natural killer (NK) cell population, observed both systemically and locally (tumor infiltration) (Kang et al., 2015).

### 4.4 Modulation of oxidative stress in *Drosophila melanogaster* model

Studies by Subedi et al. in *Drosophila melanogaster* demonstrated that Abhraka Bhasma supplementation induces a mild pro-oxidant signal, evidenced by a significant depletion of total GSH content (31%–70%) (Subedi et al., 2018a). This appears to trigger a classic hormetic response, as this stress subsequently upregulated the adaptive antioxidant enzymes SOD and catalase (Subedi et al., 2018a). This mechanism was supported by another study from the group, which showed that *Abhraka Bhasma* increased the expression of the master stress-response genes CncC (the *Drosophila* Nrf2-equivalent) and Hsp70 (Subedi et al., 2018b). This suggests *Abhraka Bhasma* may enhance stress resilience by pre-conditioning these defensive pathways. However, the primary drawback of this entire line of evidence is the vast translational gap between the *Drosophila* model and human physiology.

## 5 Ayurvedic rationale for use of *Abhraka Bhasma* in cancer/breast cancer

In *Ayurveda*, cancer-like conditions are termed *Arbuda* (malignant) and *Granthi* (benign) (Subedi et al., 2018a; Subedi et al., 2018b; Sushruta, 2008). *Stanarbuda* (Breast Cancer) (Sushruta, 2008; Charaka, 2009) is described as a hard mass (Subedi et al., 2018b; Charaka, 2009) resulting from a *tridosha* imbalance (Sen, 2005; Sushruta, 2008; Charaka, 2009) causing abnormal muscle (*Mamsa Dhatu*) proliferation and impaired metabolism (*Agni*). Its management (Sen, 2005; Charaka, 2009; Sushruta, 2008; Vagbhata, 2012) aims to remove toxins and balancing *tridosha* (Charaka, 2009; Sushruta, 2008).

### 5.1 *Rasayana* (rejuvenation) and *Dhatu-Pushtikara* (tissue nourishment) action of *Abhraka Bhasma* in *arbuda* (malignant tumor)

*Abhraka Bhasma* is a potent *Rasayana* (rejuvenation) (41, 36, p. 109–115, 215–218; 34, p. 5–36), traditionally used to enhance *Ojas* (immunity). *Ojas* correlates with modern immunomodulation and oxidative stress management (35, 37, 2008. p. 68–71; 45, 46). It also enhances resilience and nourishes bone marrow (*majja dhatu*) (33, p. 474–488; 42), providing a basis for counteracting systemic debility in cancer (Table 3).

**TABLE 3:** Term mapping box of the Ayurvedic Rationale for *Abhraka Bhasma* in Cancer (*Arbuda*).

Ayurvedic concept	Literal meaning	Modern correlation	References
*Arbuda/Stanarbuda*	Malignant tumor/Breast cancer	Cancer/Breast cancer	Kulala et al., 2023, Charaka, 2009
*Rasayana*	Rejuvenation therapy	Immunological resilience	Sen (2005), Sushruta, 2008, Vagbhata, (2012), Sharma et al. (2019), Kuchewar et al. (2014)
*Tridosha Shamaka*	*Dosha*-pacifier (for *Vata, Pitta, Kapha*)	Systemic Homeostasis, anti-inflammatory, immune modulation	Sen, 2005, Sushruta, 2008, Vagbhata, 2012
*Agni*	Metabolic fire	Metabolic Regulation, management of oxidative stress	Charaka, 2009, Sushruta, 2008, Vagbhata, 2012, Sawant and Mishra (2022)
*Lekhana*	Scraping/Desiccation	Anti-proliferative activity, Cytoreduction	Sawant and Mishra (2022)
*Dhatu-Pushtikara*	Tissue nourishment	Cellular/Tissue repair	Sushruta, 2008, Vagbhata, 2012
*Shothahara*	Anti-inflammatory	Anti-inflammatory activity	Acharya, 2015, Vagbhata, 2012, Bengal et al. (2020)
*Krimighna*	Anti-pathogenic	Anti-microbial	Acharya, 2015, Vagbhata, 2012, Bengal et al. (2020)
*Ojovardhak* *Vyadhikshamatva*	Immune enhancer	Immune modulation	Sen (2005)

### 5.2 *Tridosha shamaka* (dosha-pacifying properties)

*Abhraka Bhasma* pacifies *doshas* (37, p. 68–71, (Vagbhata, 2012), p. 94–98), the traditional etiological factors for *Arbuda* (cancerous growth). This property correlates physiologically with its capacity for systemic immune modulation. Furthermore, it has the ability to restore *agni* (metabolic fire) and perform *lekhana* (scraping) (Sawant and Mishra, 2022). These properties provide a traditional basis for its role in managing oxidative stress and exhibiting anti-proliferative activity against pathological accumulations.

### 5.3 *Shothahara* & *krimighna* (anti-inflammatory and anti-pathogenic)

*Abhraka Bhasma* (Mica Nanoparticles) has anti-inflammatory activity (1). It also has Antimicrobial and anti-parasitic activity (*Krimighna*) which helps eliminate the microbial, cellular, and malignant or foreign entities (38, p. 94–98, 44, 5, p. 67–69) (Table 3).

### 5.4 *Ojovardhaka* and *Vyadhikshamatva* (immune enhancer)

Arbuda (cancer) is a condition characterized by weakened immunity (Ojas). The Ayurvedic concept of *Ojas* can be understood as the collective immunological resilience of the body, which represents the optimal function of its neuro-immuno-endocrine axis. Ojas depletion in *Arbuda* (cancer) can be correlated with cancer-induced immunosuppression and high systemic inflammation. Abhraka Bhasma, which is *Ojovardhaka* (Ojas-enhancer), restores this vital essence and strengthens the body’s innate cellular defense mechanisms against malignant transformation ((Sen, 2005), p. 841–850, (Vagbhata, 2012), p. 923–933).

## 6 Clinical evidence on anticancer potential of *Abhraka Bhasma* (level 4)

On the evidence-based medicine hierarchy, the available clinical evidence for *Abhraka Bhasma* is limited to Level 4 (Case Reports) which is only hypothesis-generating and cannot be used to establish efficacy.

Case 1 Nimbalkar et al. (2024) reported a Stage III, Grade III invasive ductal carcinoma case managed with an integrative Ayurvedic regimen including *Abhraka Bhasma* (125 mg/day) and *Rasayana* formulations post-chemotherapy (Nimbalkar et al., 2024). The patient had reduced toxicities and an 11-year disease-free interval, suggesting a role in long-term control (Nimbalkar et al., 2024).

Case 2 Similarly, Bendale et al. (2022) reported complete tumour regression in a patient managed exclusively with Ayurvedic medicines that included *Abhraka Bhasma* (Bendale et al., 2022). This case highlighted the *Rasayana* and immunomodulatory attributes of the formulation, suggesting its capacity to enhance host resilience and contribute to tumour regression.

While these uncontrolled observational reports describe better quality of life, and prolonged progression-free intervals in selected patients, these do not establish clinical efficacy.

## 7 Discussion

*Abhraka Bhasma* (mica nanoparticles) exhibits dual pharmacological mechanisms. One pathway involves oxidative stress modulation (lowering GSH) (Subedi et al., 2018a) and the other, direct cytotoxicity, evidenced by dose-dependent effects on MCF-7 cells (Sreelakshmi and Nandagopalan, 2024), and genoprotection via enhanced DNA base excision repair (Kulala et al., 2023). A complementary mechanism, seen in STB-HO, operates via indirect, immune-mediated pathways. STB-HO showed no direct MCF-7 toxicity but enhanced tumor sensitivity to immune effectors, polarized macrophages to an M1 phenotype, and activated NK cells (Kang et al., 2015; Ebihara et al., 2023; Tau and Rothman, 2001). Its tumor suppression is linked to anti-angiogenic (VEGFR2 inhibition) and cytostatic (G1 cell cycle arrest) actions (Rajput et al., 2008; Cho et al., 2013; Vagbhata, 2012). This dual pharmacology parallels the Ayurvedic concepts of *Rasayana* (rejuvenation/immunity) (Tripathi, 2005; Sen, 2005; Vagbhata, 2012) and *Lekhan*a (anti-proliferation) (Sawant and Mishra, 2022). Though clinical evidence is limited to case reports (Nimbalkar et al., 2024), these suggest potential for reduced chemotherapy toxicities and prolonged disease-free intervals in breast cancer patients (Nimbalkar et al., 2024).

## 8 Limitations and future directions

### 8.1 Evidence-level classification and critical perspective

A critical appraisal of *Abhraka Bhasma*, using an adapted evidence-grading framework, classifies the entire body of evidence as low-level. Current data is restricted to Level 5 (preclinical) evidence and Level 4 (case reports) (Nimbalkar et al., 2024; Bendale et al., 2022) which is limited and serve only for hypothesis generation and are not definitive proof of therapeutic efficacy due to their uncontrolled nature. Crucially, high-level Level 1–3 evidence, particularly randomized controlled trials (RCTs), is completely absent. The primary limitation in the field is the significant gap between preclinical promise and clinical validation. Therefore, it is not ready for clinical recommendation and the efficacy of *Abhraka Bhasma* in cancer remains exploratory which highlights an urgent need for well-designed clinical trials before it can be considered for integration into evidence-based oncology.

### 8.2 Safety in women’s health and medical supervision

The safety profile of *Abhraka Bhasma* especially in women’s health is a critical consideration. No systematic studies have been conducted yet to assess the safety of *Abhraka Bhasma* during pregnancy and lactation and this leads to a significant knowledge gap. Heavy-metal accumulation and its unknown effects on fetal or neonatal health give rise to risks of unknown magnitude. Until comprehensive reproductive toxicity studies establish its safety, its use must be contraindicated in pregnant and lactating women. This precaution aligns with global pharmacological standards and helps ensure that therapeutic exploration does not inadvertently compromise maternal or neonatal health. Though we have encouraging preclinical findings, it is essential to have a clear boundary between these findings and clinical application. Self-medication, inappropriate dosing, or unsupervised chronic use creates significant safety concerns, including toxicity. Therefore, the use of *Abhraka Bhasma* must remain restricted to controlled therapeutic contexts under the strict guidance of trained healthcare professionals until comprehensive clinical safety data and standardized dosing regimens are available.

### 8.3 Future scope

Current findings on *Abhraka Bhasma* in breast cancer care are promising, but they are primarily preclinical. To bridge the gap between traditional use and evidence based clinical application, a structured and systematic research pathway is essential.

Randomized controlled trials assessing the adjunct role of *Abhraka Bhasma* to conventional therapies in enhancing quality of life, improving survival and reducing treatment toxicities is needed.

Mechanistic studies exploring molecular pathways must be pursued. Elucidating its pharmacological actions is critical to fill the gap between traditional Ayurvedic knowledge and modern oncology frameworks. Furthermore, collaborative research integrating Nanotechnology (for pharmaceutical standardization), Pharmacology (for mechanistic validation), *Ayurveda* (for clinical context) and Oncology (for rigorous trial design) opens new frontiers in cancer therapeutics.

## 9 Conclusion

*Abhraka Bhasma* (Mica Nanoparticles) offers a unique bridge between holistic framework of Ayurveda and specific pathways of modern cancer biology, which exemplifies its integrative relevance. The traditional use of the formulation as a *rasayana* (rejuvenator) and *ojovardhaka* (immune-enhancer) correlates strongly with modern preclinical findings of cytoprotection, immunomodulation and DNA repair. This suggests its valuable complementary strategy to mitigate the toxicities of conventional treatments and enhance therapeutic outcomes.

Eventhough the preclinical findings are promising a significant gap in mechanistic and clinical validation prevents its translation into evidence-based care. When used as an alternative treatment in breast cancer patients, critically, there is a complete absence of randomized controlled trials to validate its safety, efficacy, and potential drug interactions.

This gap emphazises the necessity of a responsible translational outlook. While preclinical data affirm *Abhraka Bhasma*’s biological promise, rigorous standardization and toxicity profiling, randomized trials are essential before clinical translation. These steps are essential before this ancient nanomedicine can be safely and effectively integrated into a modern oncology setting.
